# Taurine Alleviates Cadmium-Induced Toxicity via Genetically Specific Strategies in Two Strains of Gibel Carp (*Carassius gibelio*)

**DOI:** 10.3390/antiox11071381

**Published:** 2022-07-16

**Authors:** Wenjie Xu, Hongyan Li, Liyun Wu, Junyan Jin, Dong Han, Xiaoming Zhu, Yunxia Yang, Haokun Liu, Shouqi Xie

**Affiliations:** 1 State Key Laboratory of Freshwater Ecology and Biotechnology, Institute of Hydrobiology, Chinese Academy of Sciences, Wuhan 430072, China; xuwenjie@gdou.edu.cn (W.X.); lihongyan@prfri.ac.cn (H.L.); wuliyun@ihb.ac.cn (L.W.); hand21cn@ihb.ac.cn (D.H.); xmzhu@ihb.ac.cn (X.Z.); yxyang@ihb.ac.cn (Y.Y.); liuhaokun@ihb.ac.cn (H.L.); sqxie@ihb.ac.cn (S.X.); 2 College of Advanced Agricultural Sciences, University of Chinese Academy of Sciences, Beijing 100049, China; 3 The Innovative Academy of Seed Design, Chinese Academy of Sciences, Beijing 100101, China

**Keywords:** taurine, Cd exposure, strain, autophagy, apoptosis

## Abstract

Our previous studies in gibel carp (*Carassius gibelio*) have shown that cadmium (Cd) exposure elicits deleterious effects depending on the genetic background, and thus we hypothesized that mitigation via nutritional intervention may vary between strains. Therefore, two gibel carp strains (the A and F strains) were fed diets supplemented with 0% or 1% taurine for 8 weeks prior to 96 h Cd exposure, and the responses of antioxidant pathways, endoplasmic reticulum (ER) stress, autophagy, and apoptosis were investigated. The results showed that taurine supplementation had no effect on the growth performance of gibel carp. After Cd exposure, histological damage to mitochondria and ER, induction of oxidative stress and antioxidant responses, occurrence of ER stress, and apoptotic signals were observed in the livers. Upon the diet effects, taurine supplementation alleviated the ER-stress-induced autophagy and apoptosis after Cd exposure and stimulated antioxidant pathways. Regarding the difference between strains, taurine played a protective role in alleviating Cd toxicity through the antioxidant response, ER stress, and autophagy in the F strain, whereas such effects were achieved by the attenuation of apoptosis in the A strain. Taken together, our results demonstrate the potential use of taurine in the mitigation of heavy metal toxicity in aquatic organisms.

## 1. Introduction

Owing to widespread environmental pollution, the diverse hazardous impacts of exposure to toxic heavy metals on living organisms are becoming a global issue of great concern [1]. Cadmium (Cd) is one of the most abundant environmental pollutants in the biosphere, and it can be both toxic and carcinogenic [2,3]. Compared to other animals, aquatic species are vulnerable to Cd toxicity via the dietborne as well as the waterborne routes [4,5]. Therefore, aquatic toxicological evaluation of the effects of Cd has been widely investigated in teleosts under chronic or acute exposure in species such as gilthead sea bream, tilapia, yellow perch, and gibel carp (*Carassius gibelio*) [6,7,8,9].

Cadmium is reported to elicit deleterious effects via neurotoxicity, immunotoxicity, induction of oxidative stress, damage to organ structure, and cellular dysfunction [2,10]. Much effort has been made to investigate the mechanism of Cd toxicity and to develop a safe therapeutic approach to mitigating the toxic effects [1]. Some chemopreventive agents such as garlic extract containing specific organosulfur compounds have been used to protect against the toxic effects of Cd in both animal models and cell lines [11,12]. Cd exposure disrupts the cellular oxidative homeostasis [13] that is regulated by various enzymatic or non-enzymatic antioxidants. Importantly, oxidative stress and glutathione (GSH) depletion are crucial components of Cd toxicity in aquatic organisms [14]. Therefore, nutrients with antioxidant properties have been applied to ameliorate the hepatotoxicity by modulating antioxidant pathways, such nutrients include vitamin C, vitamin E, carotenoids, and selenium [1,15].

Taurine (TAU, 2-amino ethanesulfonic acid), as a semi-essential amino acid, is a derivative of a sulfur-containing amino acid that has multiple functions in fish physiology [16]. Taurine is usually supplemented as an additive in the diet of aquatic animals for the promotion of growth as well as boosting the reproduction system, immune functions, and antioxidant effects [17]. The mechanism of the antioxidant activity of taurine was reported to be associated with enhanced mitochondrial function that protects the mitochondria from excessive superoxide [18]. In addition, taurine has been considered as a promising candidate for the improvement of liver function, and it has been reported as possessing tissue protective effects in treating oxidant-induced injury [19,20]. In mammalian models, taurine has been reported to alleviate the toxic effects of copper, lead, aluminum, and cadmium [21,22,23]. Similarly, administration of taurine affected hepatic metabolism and reduced Cd contamination in red sea bream and catfish [16,24]. Nevertheless, the mechanisms underlying the ameliorative effects of taurine against Cd poisoning in teleost fish are still not fully elaborated.

Previous studies have shown that exposure to Cd caused different toxic effects in gibel carp (*Carassius gibelio*) A strain (CAS III) and F strain (CAS V), regardless of whether via the dietborne or waterborne routes [9,25]. Specifically, these two strains of gibel carp showed genetically based metabolic strategies in response to Cd toxicity, verifying the fact that differences in genetic background may be an important cause of metabolic differences between fish strains. To ascertain the potential of taurine in the prevention of Cd poisoning and to explore whether these effects would vary between the two strains, experiments were performed with taurine supplementation via the diet route in the present study. We assessed the liver functions of the two strains, because the liver is the center of intermediary metabolism and plays vital roles in detoxifying processes [26,27].

## 2. Materials and Methods

### 2.1. Experimental Procedures

**Figure 1:**
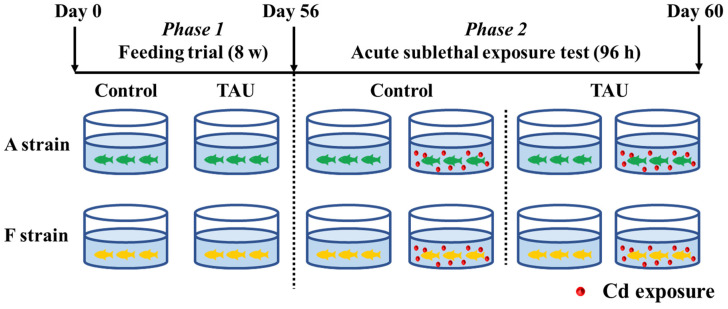
Experimental design. Control: control diet; TAU: diet supplemented with taurine.

The experimental scheme is illustrated in Figure 1. Gibel carp used in this trial were obtained from the hatchery of the Institute of Hydrobiology, the Chinese Academy of Sciences, Wuhan, Hubei, China. The healthy and uniformly sized gibel carp A (4.61 ± 0.03 g) and F (4.58 ± 0.04 g) strains were fed diets supplemented with 0% (Control) or 1% TAU for 8 weeks (Figure 1, Phase 1). Diets were formulated in the laboratory according to the procedures described by Li et al. [28]. The diet formulation and approximate composition are shown in Table 1. Therefore, four groups of fish were obtained: the A strain fed the control diet (A0), the A strain fed a 1% TAU supplemented diet (A1), the F strain fed the control diet (F0), and the F strain fed a 1% TAU supplemented diet (F1).

After the 8-week feeding trial, a challenge test was conducted with fish from each of the four groups by exposing the fish to acute waterborne Cd (11.9 mg/L) (Figure 1, Phase 2). Cd exposure was performed in a static aquarium system with continuous aeration for 96 h, with 10 fish per tank and triplicate replicates for each tank. The concentration of Cd was set based on the value shown by a preliminary experiment that identified the 96 h median lethal concentration (LC50) [25]. CdCl_2_·2.5 H_2_O was added to the water by diluting a stock solution according to methods described by Li et al. [25]. During the acute exposure experiment, water in the system was refreshed daily. This experiment was implemented following the guiding principles for the care and use of laboratory animals and was approved by the Institute of Hydrobiology, Chinese Academy of Sciences.

### 2.2. Sample Collection

At the end of the 96 h Cd waterborne experiment, fish were anesthetized with MS-222 solution (Aminobenzoate methanesulfonate, 0.06 g/L, Sigma, St. Louis, MO, USA). The livers of two fish from each tank were dissected immediately on ice, with one part frozen in liquid nitrogen and then stored at −80 °C, and one part fixed in 2.5% glutaraldehyde solution and 4% paraformaldehyde.

### 2.3. Transmission Electron Microscopy (TEM) Observation

The liver samples of the two strains were dissected into 1 mm^3^ cubes and then fixed immediately in 2.5% glutaraldehyde solution. The samples were then rinsed with 0.1 M phosphate buffer solution (pH = 7.4) three times (15 min each time). Postfixation was conducted with 1% osmium tetroxide for 2 h, and then the fixed samples were washed three times with 0.1 M phosphate buffer solution (pH = 7.4). The dehydration was performed in a graded ethanol series followed by acetone. After that, samples were infiltrated with acetone:SPI-Pon 812 resin (1:1) followed by acetone:SPI-Pon 812 resin (1:2) and SPI-Pon 812 resin. Subsequently, the samples were embedded in SPI-Pon 812 resin for 48 h at 60 °C. Ultra-thin sections (80–100 nm) were stained with uranyl acetate and lead citrate. Finally, observations were conducted using a transmission electron microscope (Tecnai G2 20 TWIN, FEI, Hillsboro, OR, USA).

### 2.4. TUNEL Analysis 

Terminal deoxynucleotidyl transferase dUTP nick end labeling (TUNEL) analyses were performed according to the procedure described by Li et al. [25]. The liver samples were fixed in 4% paraformaldehyde and then embedded in paraffin. After that, the samples were cut into 5 μm sections and deparaffinized in dimethylbenzene. The samples were dehydrated in a graded ethanol series, repaired with proteinase K, and permeabilized with Triton X-100/PBS solutions. DNA fragmentation was determined using TdT and dUTP reagents (1:9) for 2 h incubation followed by staining with 4′,6-diamidino-2-phenylindole (DAPI, 0.3 mmol/L) for 10 min. The samples were examined under a Nikon Eclipse Ti-SR inverted microscope.

### 2.5. Chemical and Biochemical Analyses

Cd concentrations in water samples were measured in accordance with the National Standards of the Republic of China (GB/T 7475-1987, Water quality determination of copper, zinc, lead and cadmium—atomic absorption spectrometry). In summary, sample digestion was conducted by adding hydrogen peroxide and concentrated nitric acid to the samples. After adding palladium nitrate, the samples were tested via inductively coupled plasma optical emission spectroscopy (ICP-OES, PerkinElmer Optima 8000, Waltham, MA, USA).

The activities of total antioxidant capacity (T-AOC), superoxide dismutase (SOD), reduced glutathione (GSH), glutathione peroxidase (GSH-Px), catalase (CAT), and the contents of malondialdehyde (MDA) were measured using commercial kits (Nanjing Jiancheng Bioengineering Institute, Nanjing, Jiangsu, China). The activity of caspase 3 (Casp3) in livers was tested using a commercial kit (Caspase 3 Activity Assay Kit, Be-yotime Biotechnology, Shanghai, China).

### 2.6. qRT-PCR Analysis

Total RNA from liver samples was extracted using TRIzol reagent (Ambion, Life Technologies, Austin, TX, USA) according to the manufacturer’s instructions. The RNA integrity and purity were assessed by agarose gel electrophoresis and NanoDrop spectrophotometer determination, respectively. The cDNA was synthesized by reverse transcription using an M-MLV First-Strand Transcriptase kit (Invitrogen, Carlsbad, CA, USA). All quantitative real-time PCR (qRT-PCR) assays were performed on a LightCycler 480 System (Roche, Jena, Thüringen, Germany). The primers used for quantitative RT-PCR are shown in Table 2. The housekeeping gene *tubulin* was chosen to normalize the relative quantification of target genes according to the methods described by Pfaffl [29].

### 2.7. Statistical Analysis

Results are presented as means ± standard errors. Normality and homoscedasticity of the data were assessed by Shapiro–Wilk and Levene tests. Two-way analysis of variance (ANOVA) was conducted with SPSS 26.0 (Chicago, IL, USA), and *p* < 0.05 was considered as a significant difference. Independent t-tests were conducted to examine the differences between pre- and post-challenge test groups. Gene expression heatmap of genes related to antioxidation, ER stress, autophagy, and apoptosis were created using heatmapper (http://www.heatmapper.ca/ accessed on 4 July 2022).

## 3. Results

### 3.1. Growth Performance

**Figure 2:**
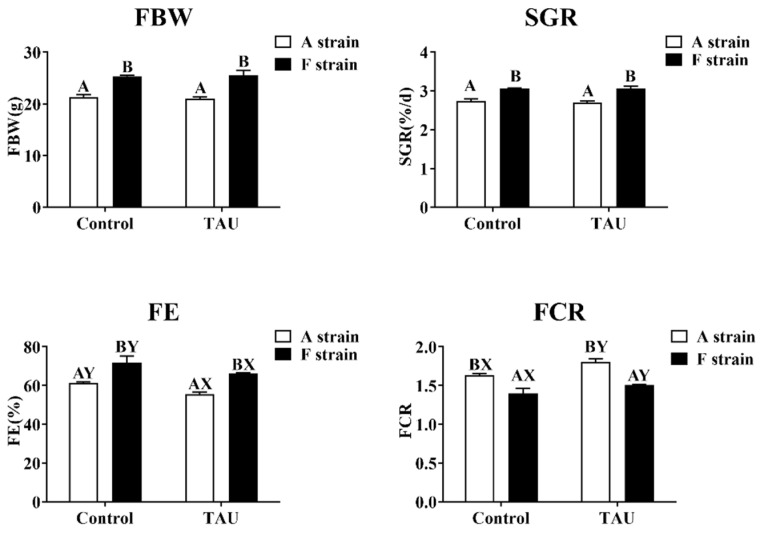
Specific growth rate and feed efficiency of two gibel carp strains fed the control diet and diet supplemented with taurine. Control: control diet, white bars; TAU: diet supplemented with taurine, black bars. FBW: final body weight; SGR: specific growth rate (%/d) = 100 × [ln (final weight) − ln (initial weight)]/day; FE: feeding efficiency (%) = (100 × body weight gain)/dry feed intake; FCR: feed conversion ratio = (100 × dry feed intake)/body weight gain. Bars with different uppercase letters (A, B) represent significant differences between the A and F strains (*p* < 0.05). Bars with different upper-case letters (X, Y) represent significant differences between the control diet group and the taurine diet group (*p* < 0.05).

No significant differences in final body weight (FBW) or specific growth rate (SGR) were observed in the two strains of gibel carp fed diets with taurine supplementation (Figure 2). However, dietary taurine supplementation significantly decreased the feed efficiency (FE) and increased the feed conversion ratio (FCR) in both strains. The F strain presented significantly higher FBW, SGR, and FE and lower FCR than the A strain (*p* < 0.05). During the experiment, the survival rate of fish was 100%.

### 3.2. Histological Observation

**Figure 3:**
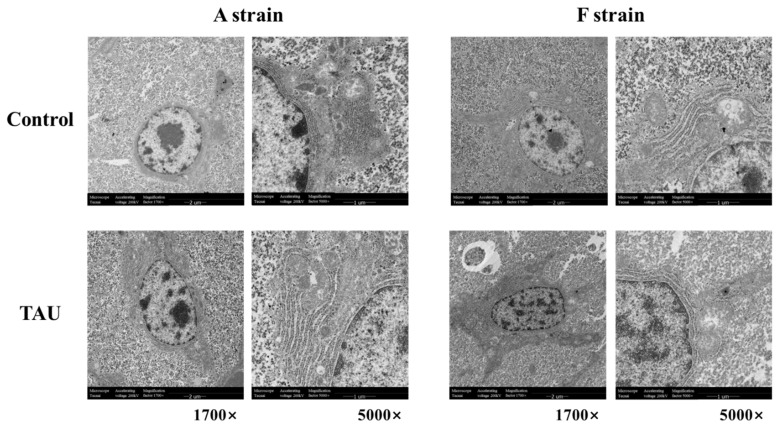
Representative histological transmission electron microscopy (TEM) images of gibel carp (A and F strains) after 96 h cadmium exposure. Control: control diet; TAU: diet supplemented with taurine.

Ultrastructural images of the liver in the two gibel carp strains exposed to Cd are shown in Figure 3. Cd exposure induced ultrastructural alterations in the two gibel carp strains, as shown by the degenerated cristae and swelling of mitochondria. Meanwhile, irregular parallel stacked endoplasmic reticulum and plaque accumulation within hepatocytes were detected by transmission electron microscopy.

**Figure 4:**
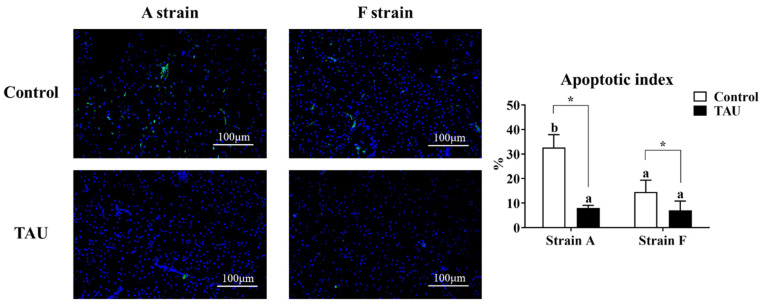
Representative DAPI and TUNEL double staining and image quantification results from the livers of gibel carp (A and F strains) after 96 h cadmium exposure. Positive apoptotic cells appear in green, and normal nuclei appear in blue. Control: control diet, white bars; TAU: diet supplemented with taurine, black bars. Bars with different lowercase letters (a, b) indicate the interaction effect and represent significant differences among groups (*p* < 0.05). Bars with * indicate significant changes between diets in the same strain (*p* < 0.05). The magnification factor is 200×, and the scale bar is 100 μm.

A terminal deoxynucleotidyl transferase dUTP nick end labeling assay (TUNEL) was conducted to assess the apoptosis index in the livers of gibel carp (Figure 4). The results showed that apoptosis signals increased significantly after Cd exposure (*p* < 0.05), while dietary taurine supplementation decreased the apoptosis index compared with the control group. Moreover, the apoptosis index in the A strain fed the control diet was the highest (*p* < 0.05).

### 3.3. Activities of the Antioxidant and Caspase Enzymes

**Figure 5:**
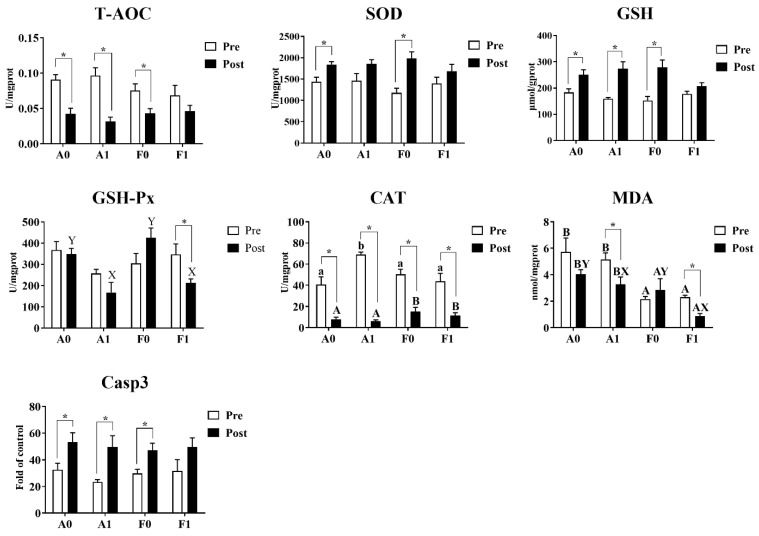
Antioxidant indices and Casp3 activity in the livers of gibel carp (A and F strains) before cadmium exposure (white bars) and after cadmium exposure (black bars). A0: A strain fed the control diet; A1: A strain fed a diet supplemented with taurine; F0: A strain fed the control diet; F1: A strain fed a diet supplemented with taurine. Bars with different uppercase letters (A, B) represent significant differences between the A and F strains (*p* < 0.05). Bars with different uppercase letters (X, Y) represent significant differences between the control diet group and the taurine diet group (*p* < 0.05). Bars with different lowercase letters (a, b) indicate the interaction effect and represent significant differences among all groups (*p* < 0.05). Bars with * indicate significant changes between before and after cadmium exposure (*p* < 0.05).

Before Cd exposure, the enzyme activities of T-AOC, SOD, GSH-Px, Casp3, and contents of GSH showed no significant variation among treatments (Figure 5). Dietary taurine supplementation significantly enhanced the activities of CAT in the A strain compared to other groups (*p* < 0.05), while MDA contents were significantly lower in the F strain than in the A strain (*p* < 0.05). After Cd exposure, no significant differences were found among groups in the activities of T-AOC, SOD, or Casp3 or in GSH content. The A strain had significantly higher GSH-Px activities and MDA contents than the F strain (*p* < 0.05). For the diet effects, dietary taurine supplementation elevated the CAT activities, whereas taurine decreased the MDA content after Cd exposure in both strains.

Cd exposure significantly inhibited the activities of CAT in both strains of gibel carp and reduced the content of MDA in the F strain. For both gibel carp strains, Cd exposure suppressed the activity of T-AOC, whereas the activity of Casp3 and the contents of GSH were elevated. However, the F strain fed the diet with taurine supplementation did not show significant differences in the activities of T-AOC or Casp3 or in GSH content. Among all groups, only the F strain given dietary taurine supplementation showed significant reduction in the activity of GSH-Px (*p* < 0.05).

### 3.4. Antioxidant Pathways and Metallothionein Levels

**Figure 6:**
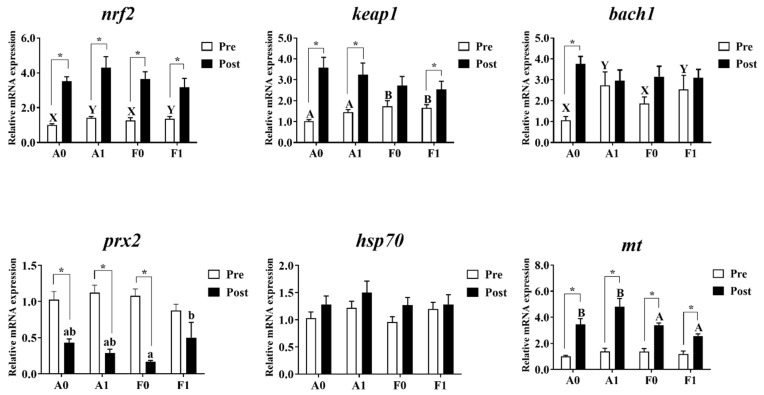
Expression levels of genes related to antioxidation and metallothionein (*mt*) in the livers of gibel carp (A and F strains) before cadmium exposure (white bars) and after cadmium exposure (black bars). A0: A strain fed the control diet; A1: A strain fed a diet supplemented with taurine; F0: A strain fed the control diet; F1: A strain fed a diet supplemented with taurine. Bars with different uppercase letters (A, B) represent significant differences between the A and F strains (*p* < 0.05). Bars with different uppercase letters (X, Y) represent significant differences between the control diet group and the taurine diet groups (*p* < 0.05). Bars with different lowercase letters (a, b) indicate a significant interaction effect and represent the differences among all the groups (*p* < 0.05). Bars with * indicate significant changes between before and after cadmium exposure (*p* < 0.05).

The expression levels of antioxidant genes and metallothionein are shown in Figure 6. Prior to Cd exposure, the gene expression levels of *prx2*, *hsp70*, and *mt* showed no significant differences among groups (*p* > 0.05). Dietary taurine supplementation significantly upregulated the expression of *bach1* and *nrf2* in both strains compared to the control group (*p* < 0.05). The F strain showed significantly higher mRNA levels of *keap1* than the A strain (*p* < 0.05).

After Cd exposure, no significant variation was observed in the mRNA levels of *bach1*, *keap1*, *nrf2*, or *hsp70* among all groups (*p* > 0.05). The A strain showed significantly higher mt mRNA levels than the F strain (*p* < 0.05). Interactions were identified in the expression of *prx2*, with the highest levels found in the F strain given dietary taurine supplementation (*p* < 0.05). Cd exposure enhanced the expression levels of *nrf2* and *mt* in both strains, while the A strain showed significant elevation of the mRNA level of *bach1* (*p* < 0.05). The expression of *prx2* was significantly upregulated after Cd exposure (*p* < 0.05), while no significant differences were observed in the F strains (*p* > 0.05). The expression of *keap1* was significantly higher after Cd exposure (*p* < 0.05), except for the F strain fed with the control diet.

### 3.5. ER Stress

**Figure 7:**
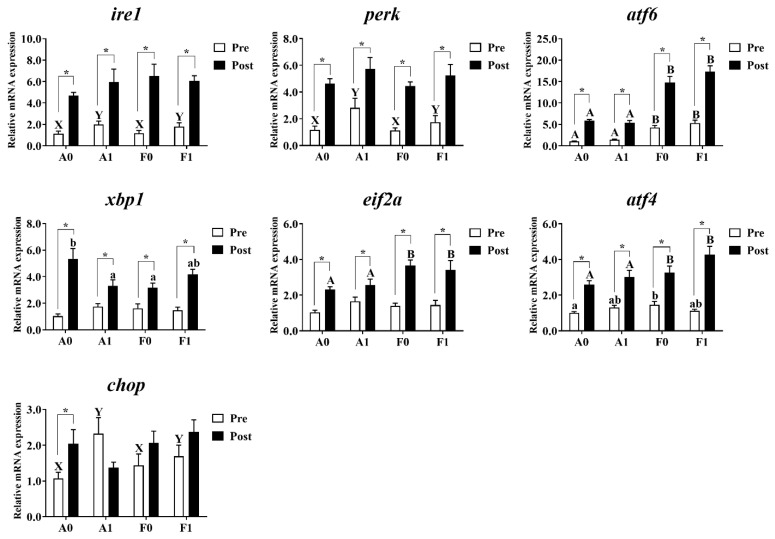
Expression levels of genes related to ER stress in the livers of gibel carp (A and F strains) before cadmium exposure (white bars) and after cadmium exposure (black bars). A0: A strain fed the control diet; A1: A strain fed a diet supplemented with taurine; F0: A strain fed the control diet; F1: A strain fed a diet supplemented with taurine. Bars with different uppercase letters (A, B) represent significant differences between the A and F strains (*p* < 0.05). Bars with different uppercase letters (X, Y) represent significant differences between the control diet and taurine diet groups (*p* < 0.05). Bars with different lowercase letters (a, b) indicate the interaction effect and represent the differences among all groups (*p* < 0.05). Bars with * indicate significant changes between before and after cadmium exposure (*p* < 0.05).

As shown in Figure 7, no significant differences were observed in the expression of *xbp1* or *eif2a* among all groups before Cd exposure (*p* > 0.05). Dietary taurine supplementation significantly upregulated the mRNA levels of *ire1*, *perk*, and *chop* in the livers of both strains compared to the control group. The F strain had markedly higher levels of *atf6* than the A strain (*p* < 0.05). Diets interacted with strains to affect the expression of *atf4* in the livers of gibel carp, with the F strain showed the highest levels among all groups (*p* < 0.05).

After Cd exposure, no significant variation was observed in the expression of *ire1*, *perk*, or *chop* among all groups (*p* > 0.05). The F strain showed significantly higher expression levels of *atf6*, *eif2a*, and *atf4* than the A strain (*p* < 0.05). Interactions were observed in the mRNA level of *xbp1*, with the highest level found in the A strain fed the control diet (*p* < 0.05). Cd exposure significantly induced higher mRNA levels of *ire1*, *perk*, *atf6*, *xbp1*, *eif2a*, and *atf4* in the livers of both strains, whereas the A strain fed the control diet had the higher *chop* mRNA levels (*p* < 0.05).

### 3.6. Autophagy and Apoptosis

**Figure 8:**
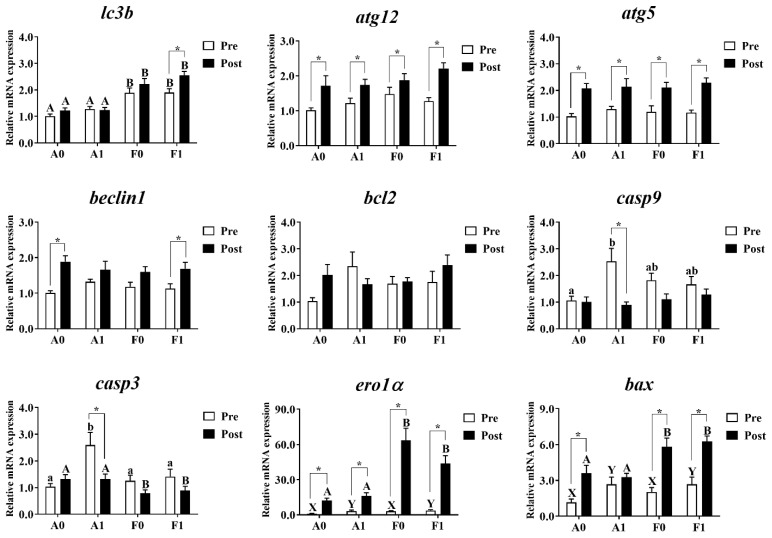
Expression levels of genes related to autophagy and apoptosis in the livers of gibel carp (A and F strains) before cadmium exposure (white bars) and after cadmium exposure (black bars). A0: A strain fed the control diet; A1: A strain fed a diet supplemented with taurine; F0: A strain fed the control diet; F1: A strain fed a diet supplemented with taurine. Bars with different uppercase letters (A, B) represent significant differences between A and F strains (*p* < 0.05). Bars with different upper-case letters (X, Y) represent significant differences between the control diet group and the taurine diet group (*p* < 0.05). Bars with different lowercase letters (a, b) indicate the interaction effect and represent the differences among all groups (*p* < 0.05). Bars with * mean significant changes between before and after cadmium exposure (*p* < 0.05).

Hepatic mRNA levels related to autophagy and apoptosis were investigated in both strains (Figure 8). Prior to Cd exposure, no significant differences were found in the expression of *atg12*, *atg5*, *beclin1*, or *bcl2* (*p* > 0.05). The mRNA levels of *lc3b* in the A strain were significantly lower than in the F strain among all groups (*p* < 0.05). Dietary taurine supplementation elevated the expression of *ero1α* and *bax* (*p* < 0.05). The diets and strains interacted to affect the expression of *casp9* and *casp3*, with the highest levels found in the A strain fed the taurine diet (*p* < 0.05).

After Cd exposure, the expression of *atg12*, *atg5*, *beclin1*, *bcl2*, and *casp9* showed no significant differences among all groups (*p* > 0.05). The A strain showed significantly higher levels of *lc3b*, *ero1α*, and *bax* and significantly lower levels of *casp9* than the F strain (*p* < 0.05). Cd exposure significantly upregulated the mRNA levels of *atg12*, *atg5*, and *ero1α* in the livers of both strains. However, the upregulated levels of *lc3b* were only found in the F strain fed the taurine diet. The increased expression of *beclin1* was found in the A strain fed the control diet and the F strain fed the taurine diet (*p* < 0.05). The A strain subjected to the taurine diet had significantly higher hepatic mRNA levels of *casp3* and *casp9*. Cd exposure induced significant upregulation of the expression of *bax* among all groups (*p* < 0.05) except for the A strain fed the diet with taurine supplementation (*p* > 0.05).

### 3.7. Heatmap Cluster Analysis

**Figure 9:**
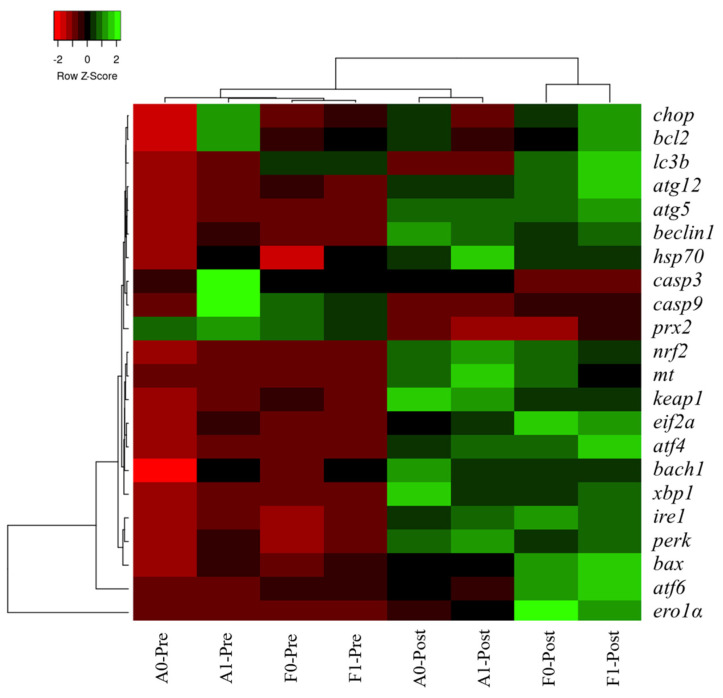
Gene expression heatmap of genes related to antioxidation, ER stress, autophagy, and apoptosis in the livers of gibel carp.

The mean values of molecular (gene expression) signatures of all treatments are presented in the clustering heatmap (Figure 9). Obvious differences were observed in the two strains before or after Cd exposure. Molecular expression of genes involved in antioxidant response, ER stress, and autophagy in the F strain after Cd exposure was not in a cluster with other treatments, especially in the F strain fed the taurine diet.

## 4. Discussion

Taurine has been reported to have beneficial effects on the growth performance of aquatic animals fed low fish meal diets in species such as black carp and white shrimp [30,31]. In the Phase 1 period of the present study, no significant effects on FBW or SGR were observed in either strain of gibel carp subjected to 8 weeks of taurine supplementation. Consistent with our results, the positive effects of dietary taurine supplementation (0, 0.5, 1.0, 1.5, and 2.0%) on growth improvement in yellowtail disappeared after six weeks of feeding, although higher final body weight was observed in fish fed a taurine diet for three weeks [32]. Dietary taurine supplementation significantly decreased FE and increased FCR in both strains. When the dietary taurine level exceeds the basic nutritional requirement, this can lead to feed intake reduction, as has been reported in Nile tilapia [33]. Therefore, the effects of dietary taurine supplement on growth of aquatic animals may be dose or time dependent.

As a semi-essential amino acid, taurine has physiological functions in the antioxidant and anti-apoptosis responses. Being a non-essential heavy metal, Cd exerts its effects and causes damage to tissues primarily through peroxidation and apoptosis [34]. Reports have shown that dietary taurine supplementation could mitigate Cd toxicity in catfish and red sea bream [16,24]. In the present study, histological observations showed that 96 h Cd exposure caused degenerated cristae, swelling of mitochondria, and irregular parallel stacked endoplasmic reticulum, and plaques in the cytoplasm of hepatocytes of both strains, suggesting that Cd damaged the mitochondria and endoplasmic reticulum in the liver cells of gibel carp. Meanwhile, Cd triggered apoptosis signals, as shown by the TUNEL results. Nevertheless, the apoptosis index was significantly lower in both strains fed diets with taurine supplementation compared to the control groups. Therefore, dietary taurine supplementation could apparently mitigate Cd-induced hepatic damage in gibel carp as in catfish and red sea bream [16,24]. Metallothionein (mt) is considered as a biomarker in the Cd detoxification process, as it can combine with Cd to form a Cd-MT complex [35]. In the present study, hepatic mRNA levels of *mt* increased significantly after Cd exposure, indicating that Cd triggered the protective proteins to counteract the damage to the liver. To further elucidate the potential protective effects of taurine in gibel carp against Cd toxicity, we investigated the antioxidant response, ER stress, autophagy, and apoptosis.

Induction of oxidative stress is one of the toxicological mechanisms involved in heavy metal stress in fish, where the production of ROS (reactive oxygen species) causes oxidative damage to cells. Previous studies have shown that the hepatic enzyme activity of SOD increased significantly in rainbow trout after 7 days of waterborne Cd exposure [36]. Meanwhile, 21 days of waterborne Cd exposure enhanced the hepatic enzyme activity of SOD in catfish [37]. In the present study, Cd exposure elevated the SOD activity in gibel carp fed the control diet. However, no significant differences were found in hepatic SOD activities in fish fed diets with taurine supplementation, implying the protective role of taurine against Cd exposure in gibel carp. MDA is considered as a biomarker of the lipid peroxidation level under oxidative stress [38]. Dietary taurine supplementation significantly decreased MDA levels in the liver, which is consistent with the results for hepatic SOD activity. Cd exposure suppressed the enzyme activity of CAT in the livers of both strains, but the F strain showed higher levels than the A strain. Moreover, Cd exposure inhibited the activities of T-AOC while elevating the activity of Casp3 and the contents of GSH. However, no significant differences were observed in the activities of T-AOC or Casp3 or in the content of GSH in the F strain fed the taurine diet. Additionally, even though the activity of GSH-Px showed no variation among groups, the lowest level was found in the F strain fed the diet with taurine supplementation. Overall, taurine may exert its protective function against Cd poisoning more efficiently in the F strain than in the A strain.

Nuclear factor erythroid 2-related factor 2 (nrf2) is a key transcriptional factor involved in the regulation of the cellular antioxidant response [39]. Nrf2 regulates downstream antioxidant-related genes such as *keap1*, *prx2*, *bach1*, and *hsp70* to alleviate oxidative stress in organisms [40]. In the present study, the nrf2 signaling pathway was activated, as indicated by upregulation of *nrf2* mRNA levels in both strains fed the taurine supplemented diet. In zebrafish, the nrf2 pathway demonstrated protective effects by mitigating Cd-induced cellular oxidative damage [41]. Before Cd exposure, the expression levels of *nrf2* and *bach1* were significantly higher in both strains fed diets with taurine supplementation than in the control groups, indicating that dietary taurine could enhance the antioxidant potential of gibel carp, while such beneficial effects were not observed after the Cd exposure. Moreover, Cd exposure downregulated the expression levels of *prx2*, except in the F strain fed the diet with taurine supplementation. Taken together, the results suggest that taurine had a protective role against Cd-induced damage in both strains, especially in the F strain.

The endoplasmic reticulum is a dynamic organelle that is responsible for folding and assembly of proteins [42]. ER stress and its downstream signaling pathways play a crucial regulatory role in response to heavy-metal-induced toxic effects [43]. Previous studies had indicated that Cd waterborne could induce ER stress in both strains of gibel carp [25]. In the present study, the expression levels of the ER-stress-related genes *ire1*, *perk*, *atf6*, *xbp1*, *eif2α*, and *atf4* were increased after Cd exposure. In other words, all branches of the regulatory pathways of PERK-eIF2a-ATF4, IRE1-XBP1, and ER stress transducers ATF6 were induced after Cd exposure, suggesting the occurrence of ER stress in gibel carp exposed to Cd. The phosphorylation dependence of PERK induces dissociation of Nrf2/Keap1 complexes, thereby triggering the transcription of downstream genes involved in antioxidant pathways [44]. The expression level of *perk* had a variation trend similar to that of *nrf2*. Meanwhile, hepatic histological alterations such as swelling of mitochondria and irregular parallel stacks of ER were triggered by Cd exposure, observations that confirmed the ER stress in gibel carp.

Autophagy refers to a catabolic process in which cytoplasmic constituents and organelles in the lysosome are degraded to maintain homeostasis as an adaptative response to stressful conditions [45]. Autophagic pathways can be triggered through induction of ER sensors under long-lasting ER stress [46]. It has been reported that Cd exposure could cause such a stress response, eliciting ER-stress-mediated autophagic and apoptosis processes in both strains of gibel carp [9,25]. Moreover, the formation of autophagosomes requires two ubiquitin-like conjugation pathways: one involves the formation of the multimeric complex of ATG5-ATG12-ATG16 conjugation; the other results in the conjugation of phosphatidylethanolamine (PE) to LC3b for the expansion of autophagic membranes [45,47]. In the present study, Cd exposure upregulated the hepatic mRNA levels of *atg5* and *atg12* in both strains regardless of the diet effect, suggesting that autophagic processes may be triggered by the increasing level of the ATG5-ATG12 complex. The mRNA levels of *lc3b* were only elevated in the F strain fed with the taurine diet, implying that more conjugation pathways were stimulated in the F strain. Thus, stronger autophagy may have been triggered in the F strain fed the taurine diet. Furthermore, Beclin-1 is a critical regulator of autophagy, because it participates in the formation of autophagosomes [48]. The transcriptional levels of *beclin1* were increased in the A strain fed the control diet and the F strain fed the taurine diet. Taken together, the results suggest that Cd exposure induced the autophagic process, and stronger autophagy responses were observed in the F strain fed the taurine diet.

Autophagy may play a protective role in cell survival, and extensive autophagy may trigger apoptosis as an independent pathway of cell death [49]. Apoptosis is also known as a cellular biomarker of metal-induced physiological alterations in aquatic animals [50]. Cd exposure was reported to induce apoptosis in topsmelt, purse red common carp, and gibel carp [25,51,52]. Apoptosis can be triggered by three main pathways, one of which is upstream caspase activation and includes the enzymes Caspase 9 and Caspase 3 [53]. In the present study, the transcriptional levels of *casp9* and *casp3* were inhibited in the A strain fed the diet supplemented with taurine. The mRNA levels of *casp3* were not consistent with the Casp3 activities, possibly due to a feedback response; a similar result has been reported in *Litopenaeus vannamei* [54]. Meanwhile, the apoptotic index in the A strain after Cd exposure was higher than in other groups as shown by the TUNEL results, suggesting that Cd exposure caused higher levels of apoptosis, and dietary taurine supplementation manifested its antioxidant effects through the regulation of the caspase gene in the A strain. In the apoptotic process, bcl2 is a member of the anti-apoptosis protein family, while bax and ero1α have opposite functions [47,55]. In the present study, no significant variation in *bcl2* expression was found after exposure to Cd, while the expression of *ero1α* was significantly elevated, indicating that apoptosis was induced by Cd exposure. The mRNA levels of *bax* increased after Cd exposure in all groups, but unaltered mRNA levels of *bax* were found in the A strain fed taurine, implying that dietary taurine supplementation alleviated the Cd toxicity by attenuating apoptosis in the A strain compared to the F strain.

Hierarchy cluster heatmap analysis showed that significant differences were observed in the two strains before or after Cd exposure, which verified the effects induced by Cd exposure as mentioned above. Expression levels of genes involved in antioxidant response, ER stress, and autophagy in the F strain post Cd exposure was not in cluster with other treatments, especially in the F strain fed the taurine diet, which was in line with previous results. Differential responses between the A and F strains of gibel carp were investigated in our previous studies owing to their genetic differences produced by selection [9,25]. The A strain was produced from eggs of gibel carp D strain and the sperm of gibel carp A strain, while the F strain was produced by the eggs of gibel carp E strain via stimulation with blunt snout bream sperm [56,57]. Therefore, a partial genome from the blunt snout bream may have been introduced into the genome of the F strain; this may have caused genetic differences between the A and F strains that led to differential genomic expression between the two strains upon Cd exposure. In the present study, even the growth performance was not significantly improved by dietary taurine supplement, but the detoxication of taurine might help to increase the survival rate of fish and raise fish quality, thereby improving the economic benefits.

## 5. Conclusions

Our study found that Cd exposure induced damage and oxidative stress in the livers of both strains of gibel carp, thereby triggering the occurrence of ER stress and the downstream responses of autophagy and apoptosis. Dietary taurine supplementation had no significant effect on the growth performance of gibel carp but did alleviate the Cd toxicity in both strains via specific genetic pathways. Dietary taurine played a protective role in mitigating Cd toxicity in the F strain through the antioxidant response, ER stress response, and autophagy, while in the A strain taurine alleviated cadmium toxicity by attenuation of apoptosis. In conclusion, the present study has provided evidence for the use of taurine in intervention or therapy for Cd poisoning in fish; thus, providing useful information for selective breeding in aquaculture.

## Figures and Tables

**Table 1:** Ingredients and proximate composition of the experimental diets (g/kg).

Ingredients	Control	TAU
White fishmeal ^1^	100	100
Wheat gluten	100	100
Soybean meal ^2^	170	170
Rapeseed meal ^2^	170	170
Fish oil	33	33
Soybean oil	33	33
Wheat flour	250	250
Taurine	0	10
Vitamin premix ^3^	3.9	3.9
Choline chloride	1.1	1.1
Mineral premix ^4^	50	50
CMC	30	30
Cellulose	59	59
Chemical composition (g/kg)		
Crude protein	340.5	344.0
Crude lipid	82.5	80.2
Ash	74.8	74.6
Moisture	79.3	88.1

^1^ Fish meal was purchased from American Seafood Company, Seattle, Washington, USA. ^2^ Soybean and rapeseed meal were purchased from Coland Feed Co. Ltd., Wuhan, Hubei, China. ^3^ Vitamin premix (mg/kg diet): vitamin A, 1.65; vitamin D, 0.025; vitamin E, 50; vitamin K, 10; ascorbic acid, 100; thiamin, 20; riboflavin, 20; pyridoxine, 20; cyanocobalamine, 0.02; folic acid, 5; calcium pantothenate, 50; inositol, 100; niacin, 100; biotin, 0.1; cellulose, 645.2. ^4^ Mineral premix (mg/kg diet): NaCl, 500; MgSO_4_·7H_2_O, 8155.6; NaH_2_PO_4_·2H_2_O, 12,500.0; KH_2_PO_4_, 16,000.0; CaHPO_4_·H_2_O, 7650.6; FeSO_4_·7H_2_O, 2286.2; C_6_H_10_CaO_6_·5H_2_O, 1750.0; ZnSO_4_·7H_2_O, 178.0; MnSO_4_·H_2_O, 61.4; CuSO_4_·5H_2_O, 15.5; CoSO_4_·7H_2_O, 0.5; KI, 1.5; corn starch, 753.7.

**Table 2:** Sequences of the primers used for qRT-PCR analysis in gibel carp.

Gene	Acronym	Prime Sequence	Amplicon Size (bp)	Accession No.
Tubulin	*tubulin*	TCCTTCAACACCTTCTTCAGTGAGAC	134	JX4135181
		AGCTGCTCAGGGTGGAACAGC		
Nuclear factor [erythroid-derived 2]-like 2	*nrf2*	CCCTTCACCAAAGACAAGCA	128	MG759384
		TTGAAGTCATCCACAGGCAG		
Kelch-like ECH-associated protein-1	*keap1*	CTCACCCCCAACTTCCTGCAG	150	MG759382
		GATGAGCTGCGGCACCTTGGG		
CNC homolog 1	*bach1*	TGGAGCGCAGGAGCTTTCGAG	98	XM_026282740
		AGTGGGGTTTGGTCGGCTGTG		
Peroxiredoxin 2	*prx2*	AGGTCATCGCTGCTTCCACCG	90	XM_026211451
		TGTTCATGGAGCCCAGGCCAC		
Heat shock protein 70	*hsp70*	CTCAACAAGAGCATCAACCCAG	155	JN006055.1
		ATGACTCCACCAGCCGTTTC		
Metallothionein	*mt*	AACTTGTTCGTCTGTGCTGG	93	XM_026230631.1
		GAGAACAACAGGGAGGTCGT		
Activating transcription factor 6	*atf6*	TGCAGGTGTATTACGCCCCTCAC	176	XM_026290872.1
		GTAATTCATAGCTGGCAGGACCAC		
Eukaryotic translation initiation factor 2A	*eif2a*	AGCTGCCAAAGAACGGCCCCATT	226	XM_026230526.1
		CAAACTTCCATCTGCCCTCTCAG		
Inositol-requiring protein-1α	*ire1*	GCGACCTTTCCTGCCTTACT	253	XM_026218282.1
		AGTCTCCTGTTTGGACAGCG		
X-box-binding protein 1	*xbp1*	CATCTACACCAAACCCACCGA	264	MN852578
		CATCCAGAGTCACTGTACGCA		
Eukaryotic translation initiation factor 2-alpha kinase 3	*perk*	TGCCATCAAGAGGATCCGTCTGC	122	XM_026224076.1
		CCTGCCAAGCATTGAAGTAACGG		
Activating transcription factor 4	*atf4*	CAGCCGAGAGATCCGCTATC	215	XM_026260813.1
		GATGAGCCCCTTACTGGACG		
DNA damage-inducible transcript 3 protein	*chop*	ACCACTCCTCGCTGACAGA	88	XM_026265784.1
		TTAGAGGCCTCGGGTCGAT		
Endoplasmic reticulum oxidoreductase 1 alpha	*ero1α*	ATGCCCAACACAAGCAACAC	129	XM_026242578.1
		TGACAACAGCGACCGAAAGT		
Microtubule-associated proteins 1A/1B light chain 3B	*lc3b*	CTACGAGCGCGAGAGAGATG	81	XM_026238789.1
		TGAGGACACGCAGTTCCAAA		
Beclin-1	*beclin1*	TGGAGAACTTGAGTCGCAGG	129	XM_026249455.1
		GCTGAGTGTCCAGATGGTCG		
Autophagy protein 5	*atg5*	GCTCTTCCGACCAGTGTCTC	188	XM_026284696.1
		AGTTGTCTGGGTGGCTCAAG		
Autophagy protein 12	*atg12*	GCTGTTGAAAGCAGTAGGTGATG	170	XM_026284438.1
		GGTCTGGTGATGGAGCAAATGAC		
Apoptosis regulator Bcl-2	*bcl2*	AAAGGATGTACCAGCGCGAA	83	XM_026237836.1
		GGCTAAGAATCTGCGTTGCG		
BCL2 associated X, apoptosis regulator	*bax*	ACCCCAGCCATAAACGTCTTGCG	214	XM_026262399.1
		GCCTTGATGACAAGCCGACAC		
Caspase 3	*casp3*	ATCATGACCAGGGTCAACCA	119	XM_026266756.1
		TACATCTCTTTGGTGAGCAT		
Caspase 9	*casp9*	ATCACAAACTACCTCAACGG	80	XM_026241892.1
		CCTCCACAGGCCTGGATGAA		

## Data Availability

Data are contained within the article.
